# The role of microorganisms in the biotransformation of psychoactive substances and its forensic relevance: a critical interdisciplinary review

**DOI:** 10.1093/fsr/owad025

**Published:** 2023-09-04

**Authors:** Eduardo G de Campos, Otávio G G de Almeida, Elaine C P De Martinis

**Affiliations:** Faculdade de Ciências Farmacêuticas de Ribeirão Preto, Universidade de São Paulo, Ribeirão Preto, São Paulo, Brazil; Department of Chemistry and Fermentation Sciences, Appalachian State University, Boone, NC, USA; Faculdade de Ciências Farmacêuticas de Ribeirão Preto, Universidade de São Paulo, Ribeirão Preto, São Paulo, Brazil; Faculdade de Ciências Farmacêuticas de Ribeirão Preto, Universidade de São Paulo, Ribeirão Preto, São Paulo, Brazil

**Keywords:** microorganisms, drugs of abuse, novel psychoactive substances, forensic toxicology, forensic chemistry, forensic microbiology

## Abstract

Microorganisms are widespread on the planet being able to adapt, persist, and grow in diverse environments, either rich in nutrient sources or under harsh conditions. The comprehension of the interaction between microorganisms and drugs is relevant for forensic toxicology and forensic chemistry, elucidating potential pathways of microbial metabolism and their implications. Considering the described scenario, this paper aims to provide a comprehensive and critical review of the state of the art of interactions amongst microorganisms and common drugs of abuse. Additionally, other drugs of forensic interest are briefly discussed. This paper outlines the importance of this area of investigation, covering the intersections between forensic microbiology, forensic chemistry, and forensic toxicology applied to drugs of abuse, and it also highlights research potentialities.

**Key points:**

- Microorganisms are widespread on the planet and grow in a myriad of environments.
- Microorganisms can often be found in matrices of forensic interest.
- Drugs can be metabolized or produced (e.g. ethanol) by microorganisms.

## Introduction

Problems related to drug abuse are still remarkably challenging for public health and safety, as they affect the lives of many people worldwide. The task of tackling drug abuse by providing strategies and solutions requires a complex approach, involving different experts (i.e. clinicians, epidemiologists, clinical and forensic toxicologists, forensic chemists, law enforcement professionals, politicians, and public health agencies). Therefore, scientists in academia, forensic laboratories, and research centres have been developing multiple and interdisciplinary strategies to provide a more exhaustive comprehension of drug problems, especially in the light of the ongoing novel psychoactive substances (NPS) phenomena. A well-known definition of NPS adopted by the United Nations Office on Drugs and Crime is that NPS are “substances of abuse, either in a pure form or a preparation, that are not controlled by the 1961 Single Convention on Narcotic Drugs or the 1971 Convention on Psychotropic Substances, but which may pose a public health threat” [1]. These substances usually mimic the psychoactive effects caused by illicit drugs (e.g. methamphetamine and cocaine) but display different chemical structures and induce severe adverse effects, which are not fully understood given that several of these drugs are “new”. On the other hand, some NPS may have been discovered decades ago and only recently emerged in the illicit drug market.

Forensic chemists and toxicologists often have to search for drugs of abuse in biological and non-biological specimens, to investigate the potential presence or absence of such substances in that sample, but it is also important to consider the possible presence of new synthetic drugs. Traditionally, forensic toxicologists mainly work with biological specimens and forensic chemists perform drug testing in non-biological specimens such as drug materials and other evidence found in crime scenes. For example, soil and solvents collected from a suspected clandestine laboratory may help in the investigation, if pure drugs are not found. More recently, in forensic research, wastewater has emerged as another interesting matrix to test for traditional illicit/licit drugs and search for NPS. Although wastewater cannot provide specific information about individual drug use, it can provide a comprehensive, community-wide understanding of exposure/intake to drugs of abuse. In this context, the knowledge about the stability of drugs in these samples is important since multiple factors may affect the overall drug stability and concentration, including the possible degradation by microorganisms.

Microbes are widely distributed on the planet, due to their ability to adapt, persist, and grow under different conditions. In this context, microorganisms have become valuable tools for forensic investigations, and several advances in molecular sciences and genomics have led to an increase in the exploration of the potential of microorganisms in forensic sciences [2]. In recent years, forensic microbiology (microbial forensics) has significantly advanced [3, 4], with the primary goal of applying well-established microbiological methods in a forensic context, particularly in source identification, such as in cases involving bioterrorism, biocrime, outbreaks of pathogens and accidental release of toxins or bioagents [3]. In particular, some of the recent applications frequently explored and reported in the literature include human identification, soil-based location analysis, and determination of postmortem interval and cause of death. For recent reviews on these applications, several references available in the literature may be consulted [3–6]. Therefore, there are many situations in which microorganisms may occur in specimens of forensic interest to toxicological and chemical analysis.

An interdisciplinary combination of microbiology, toxicology, and chemistry can be valuable to explore and understand how microorganisms may affect licit and illicit drugs, and NPS in several forensic samples, by the combination of microbiological characterization and chemical/toxicological analysis of samples. In addition, certain microbial species can be cultivated using well-established microbiological techniques and used as *in vitro* models to study the biotransformation of novel drugs of abuse, avoiding the use of murine models, which have been restricted due to ethical and animal well-being concerns. In this work, we review and discuss the role of microorganisms in metabolizing drugs as models for metabolite identification as well as in forensic specimens focusing primarily on “traditional” illicit drugs (e.g. cocaine and methamphetamine), abused licit drugs of forensic interest (e.g. as ethanol) and NPS. In addition, other drugs of forensic interest will be briefly mentioned, such as hallucinogens (e.g. ketamine), antidepressants and antipsychotics, which are often involved in forensic cases [7].

## The role of microorganisms in the biotransformation of drugs of abuse and NPS

Biotransformation consists of the conversions suffered by chemical substances (i.e. xenobiotic or endogenous compounds) within a biological system (such as the human body) [8, 9]. When referring to biological systems, this also includes microorganisms such as bacteria and filamentous fungi [8]. Therefore, microbial biotransformation can be understood as reactions mediated by enzymes released from diverse microorganisms (i.e. bacteria and fungi), converting low-molecular-weight organic compounds into analogue compounds [10, 11], which are also mechanisms by which microbes adapt to environmental changes [12]. Several chemical reactions may occur during microbial transformations, including but not limited to oxidation, reduction, hydrolysis, condensation, isomerization, unsaturation, the introduction of heteroatoms, and more [10].

The use of microorganisms as models for biotransformation studies has been proposed since the 1970s [13, 14]. Microbial models are alternative *in vitro* strategies used in studies of metabolism [15], which have been widely explored in biotechnological processes for industrial applications [12]. The use of metabolic models in forensic toxicology is paramount, especially when dealing with NPS, since its pharmacological and toxicological properties are not fully understood. These microbial models offer several advantages, including avoidance of human or animal experiments [15], low cost [12, 15, 16], easy manipulation and maintenance of cultures [12, 15–17], screening of several strains in a simple process [12, 16, 17], the possibility of regiospecific and stereospecific reactions [16, 17] and easy incubation conditions [12, 17]. Microbial models may also reveal new metabolites [17]. In addition, microorganisms may be used as platforms for producing metabolites at an adequate level for chemical and pharmacological characterization [12, 16–19].

Over the years, several *in vivo* and *in vitro* strategies and models have been used to study drug biotransformation. The zygomycete *Cunninghamella elegans* has been primarily explored as a model for biotransformation studies [20, 21], since it can perform both Phase I (reactions typically characterized by modification or insertion of functional groups such as oxidation or hydroxylation) and Phase II (reactions typically characterized by conjugation) metabolisms [15, 22]. In *C. elegans,* the Phase I reactions reported include hydroxylation of aliphatic and aromatic compounds, *N*- and *O*-dealkylation, *N*- and *S*-oxidation [20]. Metabolic regioselectivity and stereoselectivity are also exhibited by *C. elegans*, mimicking mammal enzymatic biotransformation [16], with the presence of CYP3A4 enzymes [14]. *C. elegans* produces several drug metabolites with reduced cost and manageable culture conditions [21]. Metabolic assays based on microbial cultures are relatively easy to perform and they are not very expensive, but the main drawback is the potential difference between human and fungal metabolism, besides the lack of standardized *C. elegans* tests in most forensic and clinical laboratories [21]. For a review on *Cunninghamella* models, refer to Asha and Vidyavathi [16].

## State of the art of *in vitro* studies with licit and illicit drugs and NPS

### Stimulants

It has been shown that cocaine and its monoesters can potentially be metabolized by bacteria that present esterases [23]. In fact, *Rhodococcus *sp. MB1 can use cocaine as source of carbon and nitrogen, converting cocaine into ecgonine methyl ester and benzoic acid, catalyzed by the enzyme cocaine esterase [24]. Cocaine esterase was also identified in *Pseudomonas maltophilia* MB11L [25]. Moreover, bacterial carboxylesterases (namely PnbA1 and PnbA2) were purified from cultures of *Bacillus subtilis* subsp. *subtilis* 168 and *Bacillus licheniformis* ATCC 14580, respectively [26]. These enzymes catalyzed the hydrolysis of cocaine into benzoylecgonine and methanol, similarly to human liver carboxylesterase hCE1 [26]. On the other hand, *Pseudomonas fluorescens* MBER and *Comamonas acidovorans* MBLF were also able to biotransform cocaine [27]. The hydrolysis of cocaine into ecgonine methyl ester and benzoic acid was catalyzed by a cocaine esterase expressed in *C. acidovorans* MBLF. In contrast, *P. fluorescens* MBER enzymes hydrolyzed ecgonine methyl ester into ecgonine, converted ecgonine into pseudoecgonine, and converted pseudoecgonine into pseudoecgonyl-CoA [27]. The moulds *Penicillium rubrum, Aspergillus niger, Penicillium* spp., and *Aspergillus* spp. were able to grow on non-sterile cocaine powder, and showed ability to degrade cocaine [28].

The microbial biotransformation of amphetamines has also been explored. Strains of *Cunninghamella echinulata* were able to metabolize *N*-propylamphetamine to 10 compounds, including amphetamine [29]. Amphetamine itself and other analogues were shown to undergo biotransformation by *Mycobacterium smegmatis,* with the *N*-alkyl group exerting an effect in the biotransformation mechanism [30]. Fungi belonging to the *Cunninghamella* genus can also metabolize the amphetamine analogues 4-ethoxyamphetamine, 4-propoxyamphetamine, 4-benzyloxyamphetamine, and 4-methoxyamphetamine through *O*-dealkylation and *N*-acetylation [31]. The biotransformation of other methoxyamphetamines was also investigated as well using *C. echinulata* [32, 33]. Additionally, *C. echinulata* has been shown to metabolize the methylenedioxy amphetamine derivatives 3,4-methyl​enedioxy​methamphetamine (MDMA) and 3,4-methyl​enedioxy​amphetamine (MDA), [34]. The biotransformation of MDMA mediated by *C. echinulata* led to the formation of MDA, 3,4-methylenedioxybenzyl methyl ketoxime and 3,4-methylenedioxybenzyl methyl ketone. Interestingly, MDA is a well-known human metabolite of MDMA and 3,4-methylenedioxybenzyl methyl ketoxime and 3,4-methylenedioxybenzyl methyl ketone have been previously identified as MDA metabolites in some mammals. In addition, a specific fungal metabolite called *N*-Acetyl-3,4-methylenedioxyamphetamine (NAcMDA) was also identified from both MDMA and MDA [34].

### Opioids

Several opioids can undergo microbial biotransformation mediated by a wide diversity of microorganisms. It has been demonstrated that *Pseudomonas putida* M10 isolated from industrial waste presented the ability to metabolize morphine and codeine [35, 36].

In *P. putida* M10, morphine dehydrogenase catalyzes the oxidation of morphine and codeine into morphinone and codeinone, which are further converted by the enzyme morphinone reductase into hydromorphone and hydrocodone, respectively [36–39]. In *P. putida* M10, the formation of 14-hydroxymorphine and 14-hydroxymorphinone was observed, besides the conversion of hydromorphone into dihydromorphine [37, 38]. The enzyme (3–17)-hydroxysteroid dehydrogenase from *Pseudomonas testosteronii* was also reported to be an effective substitute for morphine hydrogenase in the bacterial biotransformation of morphine into morphinone, an intermediate of the conversion into hydromorphone [38]. Another bacterial biotransformation described for morphine is the hydroxylation to 14-hydroxymorphine by *Arthrobacter *sp. [40].

Codeine undergoes biotransformation by *P. putida* M10, leading to many metabolites, including codeinone, hydrocodone, dihydrocodeine and 14β-hydroxycodeine [41]. It has also been reported that bacteria belonging to the *Streptomyces* genus can metabolize codeine into norcodeine [42, 43]. The biotransformation of codeine into hydroxylated metabolites (i.e. 14-hydroxycodeine, 14-hydroxycodeinone, 14-hydroxy-7,8-dihydrocodeine, and 14-hydroxy-7,8-dihydrocodeinone) was observed in *Rhizobium radiobacter* R89–1, a Gramme-negative bacterium isolated from soil [44]. The same bacterial species also produced 14-hydroxymorphine from morphine [44]. The cyanobacterium *Nostoc muscorum* can also metabolize codeine into 6-acetylcodeine, oxycodone, norcodeine, and morphine [45]. In the presence of oxymorphone, *P. putida* M10 led to the biotransformation of this opioid into the compound oxymorphol [37]. The microbial degradation of heroin has also been demonstrated. It has been proposed that *Rhodococcus *sp. H1 converts heroin into 6-monoacetylmorphine (6-MAM), which is further metabolized into morphine [46]. The alkaloid thebaine, naturally occuring in the plant *Papaver somniferum,* is an intermediate of the biosynthesis of morphine [47], which has also been described as a source of codeine and morphine through biotransformation mediated by *Bacillus *sp. FAR [48, 49].

In addition to the biotransformation of opioids by bacteria, fungi can also mediate the metabolism of opioids. *N*-demethylation of codeine has been reported in *C. echinulata* [43, 50, 51]*, Cunninghamella bainieri* [43, 51, 52], *Cunninghamella bertholletiae*, and *Cunninghamella blakesleeana* [43, 51]. *N*-demethylation and reduction at C-6 of oxycodone was carried out by *C. echinulata, Helicostylum piriforme, Trametes sanguinea,* and *Curvularia lunata* [50]. In the case of hydrocodone, it has been reported that the occurrence of *N*-demethylation and reduction at C-6 was carried out by *C. echinulata, H. piriforme, T. sanguinea*, *C. lunata*, and *Trametes cinnabarina* [50]. On the contrary, morphine and oxymorphone exhibited no biotransformation after incubation with *C. echinulata, H. piriforme, T. sanguinea*, *C. lunata*, *T. cinnabarina,* or *Sporotrichum sulfurescens* [50]. Fungi belonging to *Cylindrocarpon didymium* species were shown to convert morphine into 2,2′-bimorphine through oxidative reaction and metabolize hydromorphone, 6-acetylmorphine and dihydromorphine [53]. Mitragynine is another drug that exhibits opioid-like effects, and its biotransformation has been investigated in the fungus *Helminthosporum *sp., with the report of two main metabolites, identified by the authors as mitragynine pseudoindoxyl and hydroxy mitragynine pseudoindoxyl [54].

### Cannabinoids

Cannabinoids are prone to extensive biotransformation. A complete review on cannabinoids’ biotransformation by mammal and microbial species was published by Akhtar et al. [55]. One of the early reports on microbial biotransformation of cannabinoids was published by Binder and Meinsenberg [56], who reported that out of 163 bacterial and fungal strains studied, 51 attacked the Δ^9^-tetrahydrocannabinol (Δ^9^-THC) molecule primarily by hydroxylation [55, 56]. The biotransformation of Δ^9^-THC, Δ^8^-tetrahydrocannabinol (Δ^8^-THC), cannabidiol (CBD), and cannabinol through the partial oxidation of the n-pentyl side chain by *Syncephalastrum racemosum* ATCC 18192 and *Mycobacterium rhodochrous* ATCC 19067 have been demonstrated [55, 57, 58]. Moreover, hydroxylation of Δ^9^-THC has been described in *Fusarium nivale, Gibberella fujikuroi*, and *Thamnidium elegans* [55, 59]. The formation of hydroxylated derivatives of 8-oxo-Δ^9^-THC was observed in *C. blakesleeana* cultures [55, 60]. In another study on Δ^9^-THC biotransformation by 206 bacterial strains (mainly *Rhodococcus, Mycobacterium, Gordonia,* and *Dietzia*), metabolites with high polarity were detected, with maximum activities reported for *Mycobacterium *sp. ENZHR3, *Gordonia *sp. ENZHR5 and *Dietzia *sp. ENZHR1 [61]. Furthermore, oxidation of Δ^9^-THC was carried out by *Dietzia *sp. ENZHR1, *Mycobacterium *sp. ENZHR3, and *Gordonia *sp. ENZHR5 formed two, seven, and five metabolites, respectively [61].

### Benzodiazepines

Diazepam is a benzodiazepine that undergoes microbial biotransformation, according to reports in the literature, by multiple species (e.g. *Aspergillus amstelodami, Aspergillus flavus, A. niger, Beauveria bassiana, Aspergillus ochraceus, Aspergillus terreus, Aspergillus versicolor, Chaetomium globosum, Cladosporium herbarum, Coniphora puteana, Corious versicolor, C. blakesleeana, C. echinulata, C. elegans, Fusarium oxysporum, Fusarium solani, Fusarium sp., Glyocladium roseum, Paecilomyces variotii, Penicillium brevicompactum, Penicillium chrysogenum, Penicillium cyclopium, Penicillium ochrochloron, Penicillium pinophylum, P. rubrum, Scopulariopsis brevicaulis, Stachybotrys atra, Streptomyces griseus, Streptomyces setonii, Trichoderma viride,* and *Ulocladium septosporum* [51, 62, 63]). For example, the microbial biotransformation of diazepam into nordiazepam (or *N*-desmethyldiazepam), temazepam and oxazepam has been described [62]. Besides, *Streptomyces rimosus, C. bainieri,* and *C. bertholletiae* were able to transform diazepam *in vitro* [43, 51]. *Escherichia coli, Bacteroides fragilis*, and *Clostridium perfringens* can metabolize diazepam and flunitrazepam in a reinforced clostridial medium [64]. Flunitrazepam was converted to 7-aminoflunitrazepam in a mixed culture of the three species and by single cultures of *B. fragilis* and *C. perfringens* [64], with possible conversion to other metabolites. In regards to diazepam, its concentration decreased when incubated with *E. coli* and *B. fragilis*, but the metabolites nordiazepam, oxazepam, and temazepam were not detected [64].

### NPS

The biotransformation of some classes of NPS has been investigated in detail. Several synthetic cannabinoids have been studied using the *C. elegans* model. The biotransformation of the synthetic cannabinoid UR-144 was investigated in *C. elegans* using preparative high-performance liquid chromatography with a diode-array detector (HPLC-DAD) for separation of metabolites, followed by characterization with nuclear magnetic resonance (NMR) [65]. Ten metabolites were formed through reactions of mono and dihydroxylation, carboxylation, and ketone formation, alone or combination [65]. Some of those metabolites have also been reported in studies performed with human liver microsomes [65]. In another study, seven metabolites of the synthetic cannabinoid AM-1220 were described in *C. elegans* and five of these metabolites were also detected in studies with human liver microsomes [66]. These metabolites were formed through dihydrodiol formation, hydroxylation, and demethylation [66]. Using *C. elegans*, 16, 30, 26, and 25 metabolites were described for the synthetic cannabinoids 5F-PB-22, PB-22, XLR-11, and UR-144, respectively [22]. Similarities between human and fungal metabolites were reported for the four synthetic cannabinoids despite the low abundance of ester hydrolysis metabolites and the absence of glucuronic acid conjugates [22]. A good agreement between fungal and microsomal biotransformation of synthetic cannabinoids EG-018 or EG-2201 was described using *C. elegans* [67]. Additionally, other three metabolites of EG-018 and four metabolites of EG-2201 were produced by *C. elegans* [67]. The cannabinoids JWH-018, JWH-073, and AM-2201 were also proven to be metabolized by *C. elegans*, with 21, 17, and 48 metabolites of JWH-018, JWH-073, and AM-2201, respectively, being formed *in vitro*, including Phase I (for JWH-018, JWH-073, and AM-2201), and Phase II (AM-2201 only) metabolites [68]. A good agreement between fungal and human metabolites available in the literature was also described for these synthetic cannabinoids [68]. A recently emerged synthetic cannabinoid, 4F-MDMB-BINACA, was studied in the *C. elegans* model, as reported by Leong et al. [69], who described 23 metabolites of 4F-MDMB-BINACA, detecting Phase I and Phase II human metabolites [69]. Metabolites previously reported from *in vivo* studies were also formed in *C. elegans* incubations, showing the ability of this model to reproduce reactions from human biotransformation [69].

Regarding synthetic cathinones, only one study involving two synthetic and *C. elegans* model is available. Páez [70] studied the biotransformation of 3,4-methylenedioxypyrovalerone (MDPV) and methylone using two species of *C. elegans*, monitored by gas chromatography coupled to mass spectrometry (GC–MS), which allowed the elucidation of one metabolite for MDPV and four metabolites for methylone [70]. That author obtained good agreement for the metabolites produced by *C. elegans* in comparison with other mammal metabolites reported in the literature, and recommend the use of other techniques, such as liquid chromatography coupled to mass spectrometry (LC–MS) and NMR for a more comprehensive characterization of the fungal biotransformation of the cathinones [70].

Similarly to other NPS groups, the metabolism of phenethylamines belonging to NBOMe class mediated by microbes has been demonstrated. Grafinger et al. [71] used *C. elegans* to elucidate the biotransformation of 25D-NBOMe, 25E-NBOMe, and 25N-NBOMe, reporting on the formation of 14, 11, and 9 metabolites, respectively [71]. According to the authors, the main metabolic pathways observed in *C. elegans* for the three NBOMes were oxidative deamination, oxidative *N*-dealkylation combined with hydroxylation, oxidation of alcohols, mono- and dihydroxylation, carboxylation of alcohols and oxidative *O*-demethylation likely to occur combined with hydroxylation [71].

Biotransformation of tryptamines by *C. elegans* has also been explored, as reported by Grafinger et al. [15], who studied the biotransformation of *N,N*-dimethyltryptamine (DMT), 4-hydroxy-*N*-methyl-*N*-ethyltryptamine (4-HO-MET), *N,N-*diallyl-5-methoxytryptamine (5-MeO-DALT), and 5-methoxy-*N*-methyl-*N*-isoporpoyltryptamine (5-MeO-MiPT) [15]. A correlation of 63% with other models (e.g. pooled human liver microsomes, rat urine, and human urine) was found for the metabolites detected using *C. elegans* [15]. Metabolites formed through main Phase I biotransformation (hydroxylation, *N*-oxidation, carboxylation, deamination, and demethylation) were described using *C. elegans*, but no Phase II metabolites were detected [15].

### Other drugs

Other drugs have also been reported in the literature as being substrates for microorganisms. An example is the tricyclic antidepressant amitriptyline, whose biotransformation by *C. elegans* has been studied [72]. In that study, eight metabolites of amitriptyline were isolated by high performance liquid chromatography (HPLC) and elucidated by mass spectrometry (MS), NMR, and ultraviolet-visible (UV-Vis) spectroscopy, out of which four metabolites had been previously detected *in vivo* and *in vitro* [72]. Similarly, Martínez-Ramírez et al. [73] tested five different drugs (amitriptyline, metoprolol, mirtazapine, promethazine, and zolpidem) and five fungal species (*Absidia repens, Aspergillus repens, A. terreus, Gliocladium viride,* and *Mortierella polycephala*) to possibly mimic biotransformation changes that would take place postmortem. For that study, *C. elegans* was used as positive biotransformation control, and analyses were performed by GC–MS or liquid chromatography *in tandem* hybrid quadrupole-ion trap mass spectrometry (LC-QTrap-MS/MS) [73]. No metabolites were detected under incubation with *A. terreus*, *A. repens*, and *G. viride*, but metabolites of all drugs were reported in cultures of *A. repens, M. polycephala,* and *C. elegans* [73]. Those findings reiterate the effectiveness of *C. elegans* as a model for studies on the biotransformation of drugs [73]. Another study showed that fluoxetine undergoes enantiomeric biotransformation by the bacterial strain *Labrys portucalensis* F11 [74]. Analyses by high-performance liquid chromatography with a fluorescence detector (HPLC-FD) revealed a preferential degradation of (*R*)-fluoxetine over (*S*)-fluoxetine [74].

## Stability of drugs in postmortem specimens in the presence of microorganisms

Several factors can dictate the final concentrations of a drug in a postmortem specimen. During autopsies, biological fluids are usually collected and stored under the best conditions to assure sample stability (e.g. under low temperatures and with the addition of preservatives). Even though, prior to the arrival for autopsy, bodies may be subjected to several, non-controlled variable conditions [75].

The activity of cadaver-colonizing microorganisms may affect postmortem drug concentrations, metabolic profile, or both [76], adding a level of complexity to the interpretation of analytical findings for licit and illicit drugs [75], with potential to affect the determination of the cause of death [3]. Postmortem human microbiome is highly complex and diverse, and the presence of microorganisms in a postmortem specimen may be due to an authentic infection (that being an antemortem infection, connected or not to the death), contamination, commensalism, and postmortem bacterial transmigration [6, 77]. For example, femoral blood (from the region of the legs) is usually recommended for collection during autopsy because it is less readily subjected to postmortem redistribution [78]. However, postmortem bacterial infection of femoral blood may occur due to the transmigration from oral cavity, lungs, and gut or to the contamination through non-sterile collection (e.g. contamination from skin or intestinal contents) [78]. Collecting biological specimens into grey-top tubes inhibits bacterial growth but hydrolytic activity may remain for longer periods [79]. These tubes possess the anticoagulant potassium oxalate and sodium fluoride, inhibiting enzymes with glycolytic, phosphatase, and esterase activity [80]. However, hydrolytic activity remain for more extended periods, and thus keeping samples under refrigeration and performing timely analyses reduce the likelihood of bacterial hydrolysis in the samples [79].

After death, bacteria present in the lower gastrointestinal tract (GIT), oral cavity, respiratory system, and vagina migrate to other areas in the body, which are usually sterile in life [81], reaching the bloodstream after the circulation has stopped, consisting in the process of postmortem bacterial transmigration [82]. *Staphylococcus *sp. is example of a microorganism that rapidly emerges from visceral tissues by the action of its proteases whereas anaerobic bacteria usually take longer to move from the GIT [83]. In addition, *E. coli, Klebsiella pneumoniae, Pseudomonas aeruginosa, Enterococcus* spp., clostridia, and streptococci are other bacteria that undergo transmigration from GIT into blood and tissues [77]. In the moments leading to death (i.e. ceasing of circulation during the agonal stage or resuscitation procedures), a bacterial invasion is hypothesized to occur and it is known as “agonal spread” [82]. However, this phenomenon is still uncertain and under debate [77, 82, 84]. Depending on the conditions, the environment may also be a source of bacteria (e.g. soil, insects, and animals) invading a body [84].

In forensic pathology, postmortem microbial analysis is precious and can provide essential data for determining the cause and manner of death [85]. In addition, postmortem microbial analysis can be of great importance in investigating the potential activity of microorganisms that may lead to the degradation of drugs and the formation of metabolites, which could equivocally be considered as biomarkers of antemortem drug use [2]. Autolytic processes occurring postmortem in cells lead to the release of substrates for biotransformation of commensal microorganisms in the body [86]. Thus, bacteria can use some parent drugs and metabolites present in the body as substrates [84]. This bacterial activity may lead to the direct biotransformation/degradation of parent drugs, decreasing their concentrations in a postmortem specimen [81]; or it may lead to the biotransformation/degradation of Phase II metabolites (e.g. by cleavage of conjugates), increasing the concentrations of Phase I metabolites and/or parent drug, if the latter is directly conjugated during Phase II metabolism [81]. Several bacteria have been identified in human postmortem specimens (e.g*. Alcaligenes faecalis, Bacillus cereus, Bacillus *sp., *B. fragilis, C. perfringens, E. coli, Klebsiella aerogenes, K. pneumoniae, Lactobacillus *sp., *Micrococcus *sp., *Providencia *sp., *P. aeruginosa, Pseudomonas sp., Proteus mirabilis*, *Proteus vulgaris, Serratia marcescens, Shigella flexneri, Staphylococcus aureus, Staphylococcus epidermis, Streptococcus faecalis,* and *Streptococcus pneumoniae*) [84]. It is also noteworthy that *E. coli* exhibits the β-glucuronidase enzyme, which has hydrolytic activity, and this bacterium is common in GIT [79].

Fungi are also potential cadaver-colonizers [81]. The genera *Acremonium, Aspergillus, Candida, Geotrichum, Penicillium, Mucor, Trichoderma,* and *Trichosporon*, were isolated from skin, hair, mucosa, and lungs [87]. In another study, the genera *Aspergillus, Candida, Geotrichum, Mucor, Penicillium, Rhodotorula,* and *Trichosporonin* were isolated from one or more specimens of cardiac blood, lung, kidney, and liver specimens [76]. Lung and kidneys presented the highest diversity of fungal strains, attributed to the proximity and contact with the external environment [76]. *Eurotium repens* and *Eurotium rubrum* were identified in a mummified body, whilst *E. repens, Eurotium chevalieri*, and *Gliocladium *sp. were identified on the surface of skeletal remains [88].

In postmortem cases, the isolation and identification of microorganisms can provide essential data on the potential formation or degradation of xenobiotics after death, which can ultimately affect the findings and conclusions of a forensic case [3]. Postmortem microbiology scientific data have substantially increased in recent years (e.g. [6]). However, microbial biotransformation of abuse drugs in biological fluids remains little explored, with a few reports available providing the identity of microorganisms and microbial enzymes involved in the biotransformation for some specific drugs.

### Ethanol

During fermentation, bacteria and fungi can produce alcohol which could have forensic implications in postmortem or *in vivo* cases. For example, one of the most well-known implication of the gut microbiota and ethanol is related to a phenomenon called auto-brewery syndrome (ABS), a rare condition in which high levels of ethanol are produced in the GIT, by the action of bacteria, *via* fermentation of carbohydrates [89, 90]. Bacteria and fungi are the microorganisms involved in ABS and the yeasts *Saccharomyces* and *Candida* species are often reported in the literature [90, 91]. The high levels of endogenously produced ethanol may cause the typical effects after ethanol intake [89, 91]. This aspect is critical in investigating whether ABS might play a role in driving under the influence cases, which should be carefully considered [89]. However, more research and investigation on ABS are needed.

Regarding postmortem cases, it is expected that microorganisms may be present in cadavers, which has significant implications in forensic casework especially when ethanol-suspected cases are under investigation [2, 92]. Deaths due to bacterial infection or involving extensive trauma may increase the likelihood of postmortem ethanol production [93]. In contrast to other drugs, postmortem production of ethanol by microorganisms in biological specimens has been widely explored in the literature. For example, *Candida albicans, Candida parapsilosis, Corynebacterium *sp., *E. coli,* and *Candida tropicalis* were isolated from postmortem blood, likely due to sample contamination [94]. Sutlovic et al. [95] isolated *Citrobacter freundii, Enterococcus faecalis, S. marcescens,* and *Candida glabrata* from postmortem urine samples and isolated *Candida glabrata, Enterococcus faecalis, E. coli, Morganella morganii,* and *K. pneumoniae* from postmortem blood samples, which were assumed to be contaminants [95]. In a case study published in 2011, postmortem ethanol formation in a urine sample was potentially attributed to the presence of *S. aureus* due to bacteremia and *C. albicans,* associated with the patient’s diabetic and renal conditions [96]. Boumba et al. [92] observed that different microorganisms produced ethanol differentially. According to Kugelberg and Jones [93], practices such as rapid collection and storage under refrigeration can be beneficial in protecting against microbial ethanol production. An in-depth discussion on all factors influencing ethanol findings in postmortem specimens is available elsewhere in the literature [93, 97].

Ethyl glucuronide (EtG), a biomarker of ethanol consumption, has been reported as prone to bacterial degradation. In a study by Baranowski et al. [83], *E. coli, K. pneumoniae,* and *Clostridium sordellii* were isolated from authentic postmortem specimens and tested *in vitro* for the biotransformation of EtG and Ethyl sulphate (EtS). After successive analyses by liquid chromatography coupled to triple quadrupole mass spectrometry (LC–MS/MS) over 11 days, degradation by *E. coli* and *C. sordellii* was observed for EtG, whereas EtS remained stable [83]. Both *E. coli* and *C. sordellii* were tested and showed positive results for β-glucuronidase activity [83]. β-glucuronidase can hydrolyze EtG, leading to the degradation of this metabolite and therefore a decrease in its concentration [98, 99].

### Opioids

Morphine is an opioid that may be subjected to a postmortem increase in its concentrations due to the hydrolysis of morphine glucuronic acid-conjugates (Phase II metabolites) by bacterial glucuronidase enzymes [81]. Skopp et al. [100] assessed the concentrations of morphine, morphine-3-glucuronide (M3G), and morphine-6-glucuronide (M6G), in postmortem cardiac blood, and although bacteria were not isolated, the authors considered that bacteria present in the heart blood specimens may have had a partial role in the concentration changes observed. Cleavage of M3G spiked in authentic postmortem blood specimens produced morphine in four of five case samples studied by Carroll et al. [79], and microbiological tests showed no bacterial growth after 7 days of inoculation in trypticase soy broth [79]. Therefore, according to Butzbach [84], data on free and total morphine levels obtained from the analysis of postmortem specimens contaminated with bacteria may be biassed due to the instability of morphine and its metabolites, besides the possibility of inter-individual variations.

### Benzodiazepines

Nitrobenzodiazepines such as nitrazepam, flunitrazepam, and clonazepam are compounds that anaerobic bacteria can degrade to their 7-amino metabolites [101, 102]. These metabolites are also produced in human biotransformation and are pharmacologically inactive [81]. Several studies on bacterial biotransformation of nitrobenzodiazepines in biological fluids have been performed [103–105]. For example, Stevens [105] observed the degradation of clonazepam and nitrazepam in liver tissue specimens exposed to flies, suggesting that the flies carried bacteria to the tissues, which contributed to the degradation. *B. cereus, Staphylococcus epidermidis, C. perfringens,* and *B. fragilis* are some examples of bacteria summarized by Oliveira and Amorim [3] that have been associated to biotransformation of nitrobenzodiazepines. If nitrobenzodiazepines are transformed by bacteria, it is improbable that parent drugs will remain detectable in a postmortem specimen [81, 101].

### NPS

To our knowledge, there is still limited information regarding the potential microbial degradation of NPS in postmortem specimens. An example is the degradation of mephedrone by *S. aureus, E. coli, K. pneumoniae,* and *P. vulgaris* studied *in vitro* at 37°C [106]. The biotransformation products were detected in incubations with all bacterial species selected for the study, and a novel degradation product of mephedrone, 2-hydroxy-1-(4-methylphenyl)propan-1-one (HMP), was proposed. This bacterial metabolite was also observed in decomposed porcine liver and postmortem human blood, but the authors highlight that HMP might be formed by other bacteria or fungi [106]. However, due to the emergence of multiple NPS in the last decades, more investigation on the stability of NPS in postmortem specimens due to the presence of microorganisms is still needed.

### Other drugs

Gamma-hydroxybutyric acid (GHB) is another drug that had its microbial biotransformation investigated. *Clostridium* strain could metabolize GHB [107]. Elliott et al. [108] screened postmortem blood and urine specimens to identify the presence of microorganisms and their potential role in metabolizing GHB. In six postmortem blood specimens, *Clostridium* spp., *E. coli*, *P. vulgaris*, *E. faecalis,* and *Aeromonas* spp. were identified. The authors assessed the functional potential of *Aeromonas hydrophila*, *C. perfringens*, *C. sordellii*, *E. faecalis*, *E. coli*, *P. vulgaris,* and *P. aeruginosa* in producing GHB *in vitro* in blood, plasma, and urine specimens (unpreserved and preserved with NaF 0.2%), and results indicated a potential GHB formation in unpreserved blood inoculated with *P. aeruginosa* after 1 month. A possibility raised by the authors is that GHB could be formed from gamma aminobutyric acid (GABA) present in blood mediated by bacteria, since it has been shown that *Pseudomonas *sp. present enzymes able to promote such biotransformation. However, the authors highlighted that the GHB concentrations (2.3 mg/L in unpreserved blood and 1.3 mg/L in blood preserved with NaF) were lower than those usually reported in situations of endogenous formation of GHB after death, which could be as high as 30 mg/L. Therefore, the authors stated that this bacterium may not be the only source of GHB biosynthesis in blood and other factors could have contribute to their findings.

In a study by Martínez-Ramírez et al. [109], biotransformation of amitriptyline, metoprolol, mirtazapine, promethazine, and zolpidem by fungi isolated from postmortem specimens was investigated *in vitro*. Similarities between mammalian and fungal metabolites was found (e.g*. Candida sp., Geotrichum candidum*, and *Trichosporon asahii*), and metabolites formed in fungi only were also reported (e.g*. Bjerkandera adusta*, *Chaetomium sp., Coriolopsis sp., F. solani*, and *Mucor plumbeus*). The authors highlighted that some new metabolites could be potential biomarkers of fungal biotransformation. In another study by Martínez-Ramírez et al. [110], 30 fungal strains were isolated from postmortem blood, and a comparison of *in vitro* and postmortem metabolites of amitriptyline, metoprolol, mirtazapine, and zolpidem revealed a similarity between human and fungal metabolites. In addition, a fungi-specific metabolite for zolpidem has been identified. According to Martínez-Ramírez et al. [110], fungal biotransformations should be considered when analyzing decomposed postmortem specimens since, even though fungi may not be as abundant as bacteria in the human body, fungal metabolites could be found and suggest that fungi are present in the biofluid (which could ultimately affect drug levels in the sample).

## Microbial biotransformation of psychoactive substances in the environment

### Wastewater in the context of epidemiological studies

Wastewater-based epidemiology (WBE) is an approach in which drug use by a population is estimated based on drugs and metabolites levels in wastewater. This approach was proposed by Daughton [111] and reported for the first time in a study by Zuccato et al. [112]. Several studies have been published over the years exploring the analysis of traditional and novel drugs of abuse [113]. The interest in wastewater analysis in a forensic context has been growing in recent years. Drug monitoring in wastewater combines an epidemiological and analytical approach to quickly detect the potential use of illicit substances in a community served by a given wastewater treatment plant [113–115].

It is noteworthy that drug analysis in influent wastewater is subjected to multiple factors, which can dictate the final drug concentration in the sample and thus potentially affect the interpretations of these findings. The stability and fate of drugs and metabolites in the sewer are some of these factors and should always be considered. Microorganisms present in wastewater may have a role in the biotransformation/degradation of drugs and metabolites. Not only microorganisms present in wastewater but also in microbial biofilms from the sewer may have a role in the degradation of some compounds [116].

In the sewer, natural microorganisms reside in biofilms or sediments [117]. Microorganisms living in the sewer, primarily microbes belonging to the genera *Arcobacter, Acinetobacter, Aeromonas,* and *Trichococcus*, account for most of the influent wastewater microbiome [118]. In addition, the sewer microbiome is also composed by the human microbiome from domestic waste, including urine, faeces, and washing residues, derived from skin, GIT, mouth, and respiratory and urogenital systems [119]. For example, bacteria from human faeces sum up *ca.* 15%–20% of the wastewater microbiome. However, it is crucial to consider that the microbiome involved in wastewater treatment processes at the wastewater treatment plant (WWTP) is different from sewer microbiome [120]. The microbiome of WWTP is crucial for the biotransformation of micropollutants in wastewater and contributes to the quality of effluent wastewater [121]. However, the rate of microbial biotransformations during in-plant treatments may show variations amongst different WWTP microbiomes, and taxonomic diversity amongst these microbiomes could be one of the factors causing potential variations in biotransformation rates [121]. For example, in sewer, heterotrophic bacteria present faster growth and surpass other microorganisms with slow-growth rates, such as nitrifying bacteria [120]. On the other hand, in activated sludge used in wastewater treatment plants, the medium is maintained under conditions for prevailing given microorganisms, such as nitrifying bacteria [120]. These differences must be considered, especially when designing and reproducing these settings in the laboratory for biotransformation studies.

Some studies have explored how the microbiome present in wastewater may interact with drugs and metabolites. However, only a few studies on the microbial biotransformation of drugs in wastewater are available [122].

#### Ethanol

Banks et al. [123] used rising mains and gravity sewer reactors to study the stability of ethanol metabolites, EtG and EtS, in sewer. EtG and EtS exhibited instability in both reactors due to the biofilms inside the reactors that acted as degradation accelerators, but EtS was stable in wastewater only. The authors recommended further studies to explore the extent of the in-sewer biotransformation. On the other hand, they recommend not using EtG as an ethanol biomarker in wastewater due to its instability.

#### Stimulants

The stability of cocaine and its metabolites (i.e. benzoylecgonine, ecgonine methyl ester, cocaethylene, ecgonine ethyl ester, ecgonine, m-hydroxybenzoylecgonine, p-hydroxybenzoylecgonine, anhydroecgonine methyl ester, anhydroecgonine, norcocaine, and norbenzoylecgonine) was investigated in wastewater [124]. Authentic influent wastewater samples were fed to batch reactors and spiked with standard solutions of cocaine and its metabolites, with results suggesting that the main pathway for the biotransformation was hydrolysis potentially mediated by bacteria, according to the authors [124]. However, in this study the isolation and identification of microorganisms was not conducted.

In another study, the stability of cocaine, benzoylecgonine, methamphetamine, and MDMA was tested using three different sewers (i.e. rising main sewer reactor, gravity sewer reactor, and control sewer without biofilms) [116]. In the rising main reactor, microbial sulphate reduction and methane generation were detected under anaerobic conditions. In the gravity sewer reactor, aerobic, and anaerobic microbial communities were observed, and microbial sulphate reduction and methane generation were not significant. Cocaine exhibited a marked faster degradation in sewer reactors than wastewater only, and the authors assumed that those findings could be attributable to microbial activity occurring in the biofilms. Benzoylecgonine, methamphetamine, and MDMA remained stable. In addition, the formation of benzoylecgonine from cocaine was also detected in the reactors [116].

The stability of several drugs of abuse, including the stimulants cocaine, amphetamine, methamphetamine, and MDMA was investigated in a gravity sewer sediments reactor by Li et al. [125]. In this study it was observed an increased sulphate-reduction bacterial activity in sewer sediments compared to biofilms. Similar to other studies, significant degradation over time associated with microbial reactions in sediments was observed. The formation of benzoylecgonine from cocaine was also reported. Moderate biotransformation was described for the stimulants amphetamine and methamphetamine.

Influent wastewater (reaching the WWTP) is usually analyzed for forensic purposes. However, the analysis of drugs in effluent wastewater has also been studied. For example, Evans et al. [126] studied the microbial stereoselective biotransformation of amphetamine, methamphetamine, MDMA, and MDA during wastewater treatment processes, performing laboratory experiments with river water, effluent wastewater, and activated sludge. The authors observed that microbial biotransformation of the four drugs was stereoselective, with the preferential formation of S-(+)-enantiomers in activated sludge. Another finding of that study was that in river water, stereoselective biotransformation of MDMA was moderate could be attributable to potential differences between microbiome in activated sludge and the environment. In another study by Kasprzyk-Hordern and Baker [127], potential differences in microbial biotransformation occurring in wastewater treatment and in the environment are also discussed for other compounds such as ephedrines.

#### Opioids

The stability of 6-monoacetylmorphine (6-MAM) was studied in a previous work proving that microbial activity in biofilms increases degradation, and the formation of morphine from this compound was also detected [116]. In another study, the opioids codeine and methadone were shown to undergo significant microbial biotransformation in the sewer sediment reactor [125]. The formation of morphine was also observed in the reactors and potential sources could be the biotransformation of codeine or the morphine-glucuronide present in the collected raw wastewater [125].

Enriched cultures of activated sludge from a WWTP were used to study the microbial biotransformation of the opioid tramadol [128]. Ultra-high-performance liquid chromatography coupled to quadrupole time-of-flight mass spectrometry (UPLC-QTOF-MS) was applied to characterize the biotransformation products and *16S rRNA* sequencing to identify the isolates from activated sludge [128]. Bacteria belonging to the phyla *Bacteroidetes, Actinobacteria, Firmicutes, Proteobacteria, Chloroflexi,* and *Planctomycetes* were identified in original activated sludge culture and enriched cultures. A hypothesis proposed by the authors is that the genera *Bacillus, Methylobacillus, Enterobacter, Xantobacter,* and *Sphingobactreium* might play a role in the degradation of tramadol but it was not possible to associate specific bacterial strains to the biotransformation pathways described [128].

#### NPS

In the sewer sediment reactors, the synthetic cathinones mephedrone and methylone undergo significant and continuous degradation due to microbial processes in the sediments [125]. *P. putida* has been isolated from wastewater samples and incubated with the synthetic cathinone MDPV [129]. The same study assessed the stability of MDPV and the biotransformation of MDPV and other pyrrolidinophenone-type psychoactive substances in *P. putida* cultures. After incubation (66 h, 30°C), the initial MDPV quantity was reduced by ˃50%. This reduction could be associated with its use for biomass production. Transformation products of the target analytes were characterized using UPLC-QTOF-MS. Therefore, *P. putida* is a valuable model to understand part of the in-sewer transformation profile of MDPV and other pyrrolidinophenone-type compounds. However, other microorganisms should be isolated, identified and studied for a more comprehensive understanding of these transformations [129]. *In silico*, it has been suggested that some NPS (e.g. carfentanil, 4F-MDMB-BINACA, 5F-MDMB-PICA, MDMB-4en-PINACA, and mitragynine) may undergo microbial biotransformation in wastewater, potentially mediated by bacterial esterases [130].

### Soil in the context of clandestine laboratory investigation

The study of microbial biotransformation may also be applied in environmental investigations to uncover clandestine laboratories. In such places, chemical substances used in the production of illicit drugs are frequently disposed of in the soil, sewer, and general waste treatment facilities [131]. Detecting these chemicals in a suspected clandestine laboratory is a shred of crucial evidence for crime scene investigation. Therefore, proper detection is paramount. If microorganisms convert these chemicals involved in illicit drug production into new transformation products, this can affect the investigation of clandestine laboratories [132]. These microorganisms might be present in the microbiome of environments where drugs and chemicals involved in drug production are often disposed of. However, there is a lack of data on the microbial metabolic profile of drugs, precursors, and intermediates under these conditions [132].

The impact of microbial activity in soil on phenyl-2-propanone (P2P) and methylamphetaminesulphate (MAS) was investigated [132]. P2P is a precursor in several synthetic methods used in clandestine laboratories for methamphetamine production [133]. After exposition to soil microorganisms, P2P suffered biotransformation, whereas MAS remained stable [132].

In another study, Pal et al. [134] studied the stability in soil of methamphetamine, MDMA, pseudoephedrine, and *N*-formylmethylamphetamine and 1-benzyl-3-methylnaphthalene (secondary products) in soil. Methamphetamine and 1-benzyl-3-methylnaphthalene showed high stability in soil in contrast to MDMA and pseudoephedrine. Another byproduct of a route of methamphetamine synthesis, 1-(1′,4′-cyclohexadienyl)-2-methylaminopropane (CMP), exhibited low stability in either non-sterile and sterile soils, with the main transformation product of CMP being methamphetamine itself [135].

## Concluding remarks and perspectives

In this paper, the role of microorganisms as agents involved in biotransformation and degradation of traditional drugs of abuse, NPS, and other compounds of forensic interest is revisited and critically discussed through the analysis of several studies on microorganisms and drugs in contexts of interest in forensic toxicology and chemistry. To the best of our knowledge, this is the first comprehensive review on this topic covering aspects of toxicology, chemistry, and microbiology of drugs of abuse within the forensic sciences.

Microorganisms can be present in several biological and environmental specimens, and they may use drugs of abuse as substrates, leading to the biotransformation of parent drugs or human metabolites and the formation of new biomarkers. These potential microbial biotransformations can affect the interpretation of analytical findings obtained from a forensic sample, considering these microorganisms can be present in living subjects, seized materials, postmortem biological fluids, wastewater, and soil. Another critical point is that microorganisms and humans may similarly metabolize a given drug, forming common metabolites. Some microorganisms have also been successfully studied in the laboratory as metabolism models, such as the fungus *C. elegans*.

As reviewed in this paper, there is a considerable amount of studies providing data on the impact of microorganisms on the stability and biotransformation of drugs of abuse *in vitro* and in biological and environmental specimens. However, there are still gaps that need further investigation, especially emphasizing NPS. The emergence of NPS brings the need for more investigation into the potential biotransformation by microorganisms in biological and environmental species and also opens possibilities for exploring microbial models of biotransformation. Therefore, the multidisciplinary approaches combining microbiology, chemistry, and toxicology in forensic sciences opens a broad range of possibilities for further research and development.

## Authors’ contributions

Eduardo G. de Campos designed the study, performed literature research, and wrote and reviewed the manuscript. Otávio G. G. de Almeida performed literature research, and wrote and reviewed the manuscript. Elaine C. P. De Martinis coordinated the study, and wrote and reviewed the manuscript. All authors contributed to the final text and approved it.

## Compliance with ethical standards

This article does not contain any studies with human participants or animals performed by the authors.

## Disclosure statement

The authors report there are no competing interests to declare.

## Funding

This study was financed in part by the Coordenação de Aperfeiçoamento de Pessoal de Nível Superior – Brasil (CAPES) – Finance Code 001. Otávio G. G. de Almeida thanks the São Paulo Research Foundation (FAPESP) for a Ph.D. fellowship (Grant 17/13759-6). Elaine C. P. De Martinis thanks the Conselho Nacional de Desenvolvimento Científico e Tecnológico (CNPq) for a Researcher Fellowship (PQ-2, Grant 306330/2019-9).
